# Education and Training Assessment and Artificial Intelligence. A Pragmatic Guide for Educators

**DOI:** 10.3389/bjbs.2024.14049

**Published:** 2025-02-05

**Authors:** Philip M. Newton, Sue Jones

**Affiliations:** ^1^ Swansea University Medical School, Swansea University, Swansea, United Kingdom; ^2^ Institute of Biomedical Science, London, United Kingdom

**Keywords:** generative artificial intelligence (GenAI), assessment practices, academic integrity, professional regulation, cheating

## Abstract

The emergence of ChatGPT and similar new Generative AI tools has created concern about the validity of many current assessment methods in higher education, since learners might use these tools to complete those assessments. Here we review the current evidence on this issue and show that for assessments like essays and multiple-choice exams, these concerns are legitimate: ChatGPT can complete them to a very high standard, quickly and cheaply. We consider how to assess learning in alternative ways, and the importance of retaining assessments of foundational core knowledge. This evidence is considered from the perspective of current professional regulations covering the professional registration of Biomedical Scientists and their Health and Care Professions Council (HCPC) approved education providers, although it should be broadly relevant across higher education.

## Introduction

The launch of ChatGPT in November 2022 sparked an explosion of interest in new generative AI (GenAI) tools. ChatGPT is a chatbot based upon an underlying large language model (LLM). At the time of writing, ChatGPT remains the most popular tool with the greatest market share and largest user base, reaching 1 million users within days of launch [1] and currently with 200 million weekly users worldwide [2]. The most recent update to ChatGPT (Sept 2024) now allows subscribing users to select from different underlying models, including “OpenAI o1” which is specialised for advanced problem solving [3] and GPT-4o for text generation [4]. The current versions of ChatGPT can analyse images and other uploaded files, utilise external tools like Python for coding and data analysis, and are capable of real-time web searching and advanced real-time voice interaction. They are also capable of generating images, as well as the standard text, and have memory functions which allow them to remember key features about the user. Although ChatGPT remains the frontrunner, there is currently a fierce competition for share of this emerging market, with tech giants launching and developing their own products like Google Gemini, Microsoft Co-pilot and Apple AI, alongside products from new companies like “Claude” from Anthropic [5]. Most of these tools have both a free and subscription version, and the difference in performance between these is often very large, with further performance difference obtained as the underlying LLMs are updated. For example, the early free versions of ChatGPT, running GPT-3 or 3.5, tested on a range of different multiple-choice exams, scored an average of 54%. The subscription version running GPT-4 averaged 75% [6], and then the update to GPT-4o scored 94% on the UK Medical Licensing Applied Knowledge test [7], a considerable improvement in less than 2 years. These advancing/developing abilities have naturally led to discussion about the considerable opportunities and challenges generated by these tools, along with startling headlines. Here we take a pragmatic evidence-based approach [8] to summarising the current perspective on what these tools can and cannot do with current assessment methods in UK Higher Education and in practice-based training for statutory regulated professions, with a focus on biomedical scientists. We use this to generate advice for educators and trainers who wish to capture the opportunities and address the challenges afforded by GenAI. This guidance emphasises assessment in Biomedical Science, although should be applicable more broadly across higher education and in laboratory-based training.

### What is Assessment for?

Assessment is one of the defining features of a higher education provider. The definition of a university in the UK is, simply, an organisation with “degree awarding powers” [9]. The degree is based upon the university certifying the learning undertaken by that student. That certification is made almost entirely based on assessment, meaning that effective assessment is at the heart of what defines a university. Assessment is also the basis of very many aspects of other educational organisations such as the Institute of Biomedical Science (IBMS) who are a Health and Care Professions Council (HCPC) approved education provider in the UK. The Institute offers a range of education and training, plus bespoke qualifications designed for biomedical scientists who are both pre- and post-registration with the HCPC. Further, as the professional body for biomedical science in the UK, the Institute accredits both BSc and MSc programmes delivered by higher education providers. The taught curriculum of these programmes is carefully aligned with the- Quality Assurance Agency (QAA) Subject Benchmark Statement for Biomedical Science and Biomedical Sciences [10] and the Framework for Higher Education Qualifications (FHEQ) and Scottish Credit and Qualifications Framework (SCQF) for academic level [11]. Following assessment adjustments made during the COVID-19 pandemic, the Institute (in 2021) required IBMS Accredited higher education providers to return to closed book assessments and on campus, invigilated examinations from 2021 onwards. This requirement was reinstated to ensure that the knowledge and understanding of each individual student was being assessed to meet the HCPC Standards of Education and Training (SETs), specifically SET 6.3 *Assessments must provide an objective, fair and reliable measure of learners’ progression and achievement* [12]. It is essential that students studying a degree programme that can lead to statutory regulation have the appropriate underpinning knowledge and understanding to protect patient safety.

For all learners, assessment has an important place in learning itself, due to the “testing effect”: one of the most effective and evidence based approaches to improving and supporting learning is the use formative practice tests as part of the learning process [13]. This process makes use of so-called “retrieval practise” wherein prior knowledge is “brought to mind” during the learning process, as a way of enhancing integration of new knowledge with that is already known [14]. This is true whether the learner is studying taught content in the university environment or undertaking training and learning in a clinical laboratory. The iterative process of undertaking tasks, receiving feedback and using that feedback to improve supports achievement and promotes confidence in the learner. The process of regularly checking in with the learner and discussing the learning that has taken place can also provide a useful safeguard in ensuring that the piece of work is original, if the learner is able to explain it clearly and in detail.

### Different Types of Assessment for Different Levels of Learning

There are many so-called hierarchies of learning, of which Blooms Taxonomy is perhaps the most famous and well known [15]. Many of these taxonomies have flaws in the underlying cognitive science which is used to attempt to explain them, and in their practical implementation [16], but most are based on a basic truth; that knowledge and learning are cumulative. One cannot attain the so-called higher order states of learning without having a basic grasp of factual or foundational knowledge [17]. This is true perhaps in STEM (Science Technology Engineering and Maths) fields more than some other disciplines, given the large amount of technical and esoteric terminology that are needed to be able to work and learn effectively. The cumulative rather than hierarchical nature of learning is fundamentally important to understanding what makes a good assessment. Cumulative means that a learner requires knowledge of basic facts and principles before being able to work with them in applied, critical or higher order ways. Therefore, different types of assessment are more appropriate for different levels of learning, but we cannot skip the basic knowledge tests if we want to be confident that learners have achieved this knowledge before progressing safely on to higher order learning.

### Effective Assessment Design

There are many features that determine whether an assessment is effective, and there are many different types of assessment. A simple summary of these combined principles is that a good assessment is a measure of what a learner can *do*, rather than what they “know” or “understand” since these concepts cannot be objectively observed or measured [15]. Thus different types of assessment require learners to do different things. An assessment of factual knowledge might test students’ ability to identify core concepts, measured by simple multiple-choice questions, while a higher-level assessment might measure student’s ability to critically appraise the latest evidence on a topic, measured through a viva or a practical exam. Underneath that, perhaps the most important feature of assessment design is validity - does the assessment measure the thing that it is supposed to? For example, if a learning outcome is for a learner to be able to use a pipette, then a valid assessment requires them to use a pipette (rather than, say, write an essay on pipetting). Another important feature of assessment design is reliability; if the same learner took the same assessment twice under the same conditions, would they get the same mark? Would two different learners who performed the same way in an assessment both get the same mark? Reliability is important for validity, but also for other important features of assessment design such as fairness, inclusivity and the learner experience. Further, there are very important but often-overlooked features of assessment design, including cost and other practical considerations.

A good simple summary of the different types of assessment used in higher education was provided by Phil Race [18], and some of the most common types are summarised below. We will break down each different type of assessment and then discuss the ways in which the current evidence on GenAI demonstrates what these tools can and cannot do with different types of assessment, how GenAI affects validity, etc., and then how these tools might be effectively incorporated into assessment in both higher education and laboratory training. The vast majority of the research literature on this topic appears to be focused on ChatGPT, although where literature on other new GenAI tools exists, the findings appear largely similar to those found with ChatGPT [19, 20].

## Multiple-Choice Questions (MCQs)

These are a long-established assessment type which exists in multiple formats, but perhaps the most common is where the learner is given a short question, sometimes accompanied by a problem scenario, and is asked to pick a single best answer from a list of options. MCQs are traditionally associated with the assessment of lower order learning, although if written appropriately they can be used to assess higher-order learning [21] and are used this way in many disciplines, such as medicine [22]. ChatGPT has shown outstanding performance on MCQ examinations, both lower and higher-order, across a range of different disciplines including in the field of biomedical sciences [6, 23]. One of the most recent updates to ChatGPT scored 94% on the UK Medical Licensing Test, and this exceptional performance was reproduced even when the questions themselves were completely novel and had therefore not formed part of the training material for ChatGPT [7]. Importantly, many of these studies include MCQs that are partly or completely based on images, or data analysis not just theoretical knowledge. Versions of ChatGPT based on GPT-4o or later are able to read and analyse images and any text contained within them [4], meaning they can correctly answer MCQs and other exam questions that contain images or data analysis tasks [7], something that earlier tools including GPT-4 struggled with [23].

### Guidance for Educators

There is still an important place for the use of MCQs. They are an efficient way of assessing both lower and higher order learning across a broad swathe of any curriculum, with (if desired) immediate feedback for educators and students. The data above, however, suggest that all summative assessments based on multiple choice questions should be undertaken under closed book, supervised circumstances. During the rapid switch to unsupervised online exams undertaken during the COVID lockdowns, there was a considerable rise in exam cheating such that more students appeared to be cheating than not [24]. This phenomenon was even before ChatGPT, and an abundance of evidence shows that one of the main drivers for cheating is simply where there are opportunities to cheat [24, 25], meaning that using online unsupervised exams risk putting students in an impossible position of being prevented from using an easily available online tool that could gather them a very high grade, but with no effort made by the education provider to enforce that prevention.

Online exams potentially offer many cost and perceived inclusivity benefits to both educators and learners, and so the use of online invigilating/proctoring tools seems like an intuitive way to maintain these benefits while mitigating some of the assessment integrity challenges posed by GenAI. Proctoring tools are, however, associated with a poor student experience, and it is recommended to involve learners in the development of policies and processes in the use of any remote proctoring [26]. Because of these issues, the Institute has refused to accept online proctored coursework tests or examinations as part of the assessment strategy for any IBMS Accredited BSc or MSc programme.

GenAI tools can be used effectively to embed retrieval practice into learning, teaching and training. A number of studies have shown that ChatGPT is effective at generating accurate and well-designed MCQs [27–30] and these could be a simple and efficient way to harness some of the proposed opportunities to use GenAI for personalised learning. Under these circumstances, learners can use GenAI as an academic tutor that asks them questions on topics they need support with, and can give accurate and constructive explanations alongside the correct answer. The profound differences in performance between the current free and subscription versions of these tools risk exacerbating a “digital divide” between those who can and cannot afford the subscription, although it is important to contextualise this; these tools are currently much cheaper than other digital assistants and are less reliant on internet speeds [31], and the current cost for a GenAI tool subscription covering a full 3 year undergraduate programme would likely be well exceeded by the cost of recommended hard copy textbooks which arguably have a lower return on investment.

## Essays

ChatGPT shows exceptional performance when writing longer written pieces of work such as essays, producing work of comparable quality to human authors [32–34], including in the field of biomedical sciences [35]. Early reports showed that the older versions of ChatGPT would “hallucinate” references in academic writing; creating references and reference lists that looked plausible but did not actually exist [36, 37]. This issue has been reduced by linking to external tools, in particular real-time web searches, and there are now customised GenAI tools such as Jenny.AI which are specifically designed to generate academic writing. The text written by ChatGPT is often difficult for markers to distinguish from that which might have been written by students [38]. In one notable study, a team of researchers from the University of Reading “spiked” ChatGPT-generated exam answers into the marking load of academics, meaning the answers were blind marked. The ChatGPT answers were marked with equivalent grades or higher to those written by human students, and were almost never flagged as problematic [32]. The reluctance of academics to flag suspicious content may be in part because it is a challenge to independently verify whether the content has been generated by GenAI. Detection tools do exist and have been widely promoted. Contrary to some of the popular narrative, these tools are actually quite effective at detecting the raw text output from tools like ChatGPT [39, 40]. However, a main challenge with the use of these tools is the standard of evidence that they provide and the number of (or the lack of) additional pieces of evidence which can be gathered to corroborate or disprove any allegation of the learner has used AI to construct their coursework. For example, with standard “copy-and-paste” plagiarism, Turnitin is a tool which can be used to identify whether the text submitted by a learner matches the text written elsewhere on the Internet or in other published or printed works. If a match is detected, then this match, and the external source, can form the basis of a discussion between the education provider or trainer and the learner to determine how it is that such a close match has been arisen. When it comes to GenAI, there is no such “smoking gun.” AI detection tools provide a mathematical estimate of the likelihood that the text has been generated by AI but no further evidence is available from these tools. This has led to serious concerns about false allegations of misconduct against learners [41]. If an education provider wishes to pursue an allegation of plagiarism against the learner, they are likely to have to conduct some form of alternative assessment or interview, such as a viva, to determine whether the student truly does meet the relevant learning outcomes being tested in the written piece of work. In addition to these challenges, there is also an abundance of advice on YouTube for students who might wish to use ChatGPT to write their essays and then employ a variety of different tactics to cover their tracks, and these simple pieces of advice are demonstrably effective in reducing further the effectiveness of AI-detection tools [42]. Essays were already a vulnerable assessment method before the advent of ChatGPT. Traditional plagiarism has long been a concern, along with the abundance of “essay mills” which offer to write a student’s assignment for them, normally very quickly [43].

### Guidance for Educators

New GenAI tools appear to be the final straw for the validity of asynchronous essays as an assessment, unless academic writing is the specific skill being assessed (e.g., dissertations and research papers) and even then the challenges remain. Essays are commonly used as a proxy for other forms of learning and perhaps we simply need to move away from them: if we want to assess the ability of students, apprentices and graduates to become biomedical scientists, then we should focus much more on their ability to *do* biomedical science, rather than their ability to write about it. Long-form academic writing can be a useful way to learn, and to practise the construction of a critical argument in the sciences, but the assessment itself might be better in a different form. The use of oral assessments (viva voces) when testing investigating cases where a learner is suspected of having used ChatGPT to write an essay is testament to this. If the viva is itself a meaningful assessment of the learning, then perhaps a viva or professional discussion should just have been used in the first place? There is an abundance of guidance and evidence to support the use of interactive oral vivas (e.g., [44]) and these often have the added benefit of being more authentic assessments. This is why the IBMS registration training portfolio and specialist portfolio have viva elements built into the final assessment (verification or examination) to ensure that the candidate has the required knowledge, understanding and experience in a clinical laboratory to pass the assessment.

## Practical Exams

The performance of GenAI tools on practical assessment formats requires further investigation, but at the time of writing it seems that practical exams/performative tasks/competency tests are an area where GenAI could have clear benefits for assessment and assessment preparation. These include the creation of personalised learning materials, test cases, providing personalised feedback and tailored assessment tasks [45]. ChatGPT appears to function well as a simulated patient for Observed Structured Clinical Exams (OSCEs) [46] and this leads naturally to a thought that GenAI tools might function as examiners [47], especially given long-standing concerns about the reliability of human examiners in this assessment format [48]. GenAI tools also seem to offer some benefits and challenges to other practical formats such as oral and laboratory assessments, where students might use them to complete components of their assessments, making it challenging to determine whether the assessment is a valid certification of the learning of the student [19].

## Registration Training Portfolio Evidence

In the context of developing laboratory competency and demonstrating the HCPC Standards for Proficiency (SoPs) [49] to become a biomedical scientist, approaches to training that are already embedded can be useful in counteracting the nefarious use of GenAI. It is common practice in laboratory training for the trainer to demonstrate techniques, pieces of equipment and how to analyse clinical tests and patient data directly to the student, apprentice or trainee (the learner) and then ask them to explain the theoretical and practical concepts back to the trainer. Competency tests in the laboratory and direct observations of practice (DOPs) are also common methods to assess if the learner is developing their practical skills appropriately. In these scenarios where the learner is demonstrating knowledge, understanding and laboratory skills in real time, the assessment is both authentic and robust. It would be difficult for the learner to demonstrate the expected learning outcomes of such assessment tasks unless they had developed the expected level of competency and technical skill.

For written pieces of work that might be used as evidence for the IBMS registration training portfolio, or specialist portfolio, the Institute recommends using an iterative approach of reviewing drafts of work that are discussed with the learner. This process again allows the trainer to determine if the written content is well understood by the learner.

To further assist trainers during the completion of the registration training portfolio, the digital portfolio platform OneFile allows the screening of written pieces of evidence using Turnitin. Each learner will have access to the Turnitin report and it is expected that they will discuss the report with their trainer. If the Turnitin tool detects lots of sources that account for individual low scores, it is likely that the learner has researched the piece of work and has referenced the content appropriately. A high score could result from matching external sources to the reference list alone which should not cause concern, but several sentences or paragraphs of text being identified as identical to other sources constitutes plagiarism. In this case, the trainer should speak to the learner about plagiarism and academic misconduct (which they will have covered during their degree programme) and the piece of evidence would be updated and changed to remove any plagiarism.

It is essential that the learner demonstrates the required level of theoretical knowledge and application of this to clinical scenarios, real life patient data, or a technique they have performed in the laboratory. As mentioned above, ChatGPT-4o (or later versions) is able to read and analyse images with text [4], so can be used to complete data analysis tasks [7]. It is therefore vital that the trainer discusses each piece of evidence with the learner to ensure that the learner can interpret data and complete case-study based tasks in real time. Regular check-ins with learners builds confidence in their learning and also allows the trainer to gauge if they are displaying the expected level of knowledge and understanding to become a biomedical scientist. These interactions also allow the trainer to become familiar with the writing style of the learner and any sudden changes in the style, construction or wording of pieces of evidence might warrant a further discussion as to how the evidence was produced.

Regular discussions during training also effectively prepare the learner for the final assessment of the portfolio, the verification or examination. The laboratory tour conducted as part of the verification process is vital in confirming that the candidate meets the threshold standards to be awarded the IBMS Certificate of Competence and become eligible to register as a biomedical scientist. The interactive discussion, including detailed question and answer style of oral assessment in real time that is used in both verifications and examinations offers a robust way to determine if the candidate has developed their professional knowledge and laboratory skills to the required level.

## Ethical Framework for Registrants

It is important to note that as a registered biomedical scientist, the trainer or mentor must abide by the updated HCPC Standards of Conduct Performance and Ethics [50] and manage the risk associated with the learner achieving registration without having the required knowledge of the subject area, laboratory techniques or data analysis. If they have used information within their evidence that is not their own and they do not understand it. Particularly relevant to this issue are Standards 6.1 and 6.2:

6.1 You must take all reasonable steps to reduce the risk of harm to service users, carers and colleagues as far as possible.
6.2 You must not do anything, or allow someone else to do anything, which could put the health or safety of a service user, carer or colleague at unacceptable risk.

Further, the position of the Institute is very clear on plagiarism. The registration training portfolio Guidance documents for candidates and trainers [51] state: “*A plagiarism statement to confirm the portfolio is the candidate’s own work. It is important for the candidate to acknowledge the various resources used during their training and in their evidence. Any evidence of plagiarism will result in failure of the portfolio and the candidate will be required to complete a new Registration Training Portfolio.*”

If plagiarism and/or collusion is detected in the production of any piece of evidence submitted to the trainer or portfolio mentor for feedback, these pieces of evidence must be rewritten and must be the individual candidate’s own work. Any portfolio evidence that contains plagiarism, the use of GenAI platforms (including ChatGPT) or collusion will not be accepted for the final assessment (verification). If the verifier subsequently finds plagiarism, the use of GenAI and/or collusion in any evidence submitted, they will alert the Institute, the candidate will fail and have to start a new portfolio.

During the creation and collation of portfolio evidence, it might be useful to remind the learner of their responsibilities with respect to the HCPC Standards of Conduct Performance and Ethics [50]- standards summarised below:

3.1 You must only practise in the areas where you have the appropriate knowledge, skills and experience to meet the needs of a service user safely and effectively.
3.2 You must undertake additional training to update your knowledge, skills and experience if you wish to widen your scope of practice.
3.3 You must refer a service user to an appropriate practitioner if the care, treatment or other services they need are beyond your scope of practice. This person must hold the appropriate knowledge, skills and experience to meet the needs of the service user safely and effectively.
3.4 You must keep your knowledge and skills up to date and relevant to your scope of practice through continuing professional development.
3.5 You must keep up to date with and follow the law, our guidance and other requirements relevant to your practice.
3.6 You must ask for feedback and use it to improve your practice.
9.2 You must be honest about your experience, qualifications and skills.

## Fitness to Practice

The HCPC information on what an employer should do to raise a fitness to practice issue clearly states that dishonesty, fraud or abuse of trust or position are the types of issues that would be considered when raising a concern. It is important that all learners (for the registration training portfolio, specialist portfolio and the Institute’s advanced qualifications) are reminded that using other people’s work or published material without proper citations and referencing is plagiarism and amounts to dishonest and fraudulent work. Using work from other people or information from published sources without rewriting it does not fulfil the requirements of any IBMS qualification and fails to meet several sets of standards produced by the regulator for the profession and to protect the public.

### Non-Assessment Uses of AI

GenAI is clearly here to stay and therefore all graduates from different subject areas in the future will be using AI as part of their jobs and everyday life. For biomedical scientists, the development and enhancement of digital pathology tools means that AI will inevitably form part of their working lives. Therefore, it seems reasonable to expect that higher education providers should determine whether students can use AI affectively and teach them to do so. This is one of the most rapidly evolving and yet most poorly formed areas of discourse regarding AI in higher education, and one of the reasons for this is that we do not yet know what effective AI use will look like in the workplace. For example, there is an abundance of examples to show that GenAI might be well used in diagnostic or other areas of practise. A full review is beyond the scope of this paper but AI tools have been advocated as effective in areas including radiography [52] and pathology [53]. As digital pathology continues to evolve, we do not yet know what future practise will look like. It seems reasonable that we might wait to see what practise looks like before we start rushing into embedding AI training and literacy into higher education, and redesigning our assessments to bring GenAI into them. A final component of assessment design, particularly relevant for professional programmes such as Biomedical Science, is that the assessments should be authentic, reflecting how learning will be used in the real world. We do not yet know what ‘authentic’ looks like when considering the use of AI in practice.

## Summary

ChatGPT is having a profound effect on current assessment practice in higher education, and subsequently on assessment types used in work-based training, not least due its outstanding performance on many of the common assessment methods we use. This creates fears of cheating but also suggests that there may be many benefits to bringing ChatGPT into learning and assessment. One intuitive and frequent, but mistaken, assumption is that, if ChatGPT can answer basic knowledge tests then perhaps they should be scrapped and replaced with more advanced and applied assessments. Learning is cumulative, and higher order critical thinking depends on having excellent knowledge of basic facts and concepts [17]. Thus, we need to ensure that learners are taught relevant facts and concepts but are then able to demonstrate their knowledge and understanding in secure assessment formats that exclude tools like ChatGPT. The assessment of higher order learning is perhaps more problematic, unless one to one discussions are used to assess synthesis, analysis and evaluation. Clearly GenAI tools are here to stay and will likely (though not definitely) be an important part of biomedical sciences professions in the next few years. Assessments, and education and training generally, will have to remain cognisant of the developments and move with them to ensure that they reflect the authentic world of work while retaining human autonomy.

## Abbreviations

AI, artificial intelligence; GenAI, generative artificial intelligence; LLMs, large language models.

## References

[1] Statista. Selected Services Reaching 100 Million Followers 2024 (2023). Available from: https://www.statista.com/statistics/1489983/selected-platforms-services-reach-one-hundred-million-followers/ (Accessed October 21, 2024).

[2] Reuters. OpenAI Says ChatGPT’s Weekly Users Have Grown to 200 Million. Reuters (2024). Available from: https://www.reuters.com/technology/artificial-intelligence/openai-says-chatgpts-weekly-users-have-grown-200-million-2024-08-29/ (Accessed October 21, 2024).

[3] OpenAI. Introducing OpenAI o1 (2024). Available from: https://openai.com/index/introducing-openai-o1-preview/ (Accessed October 2, 2024).

[4] OpenAI. Hello GPT-4o (2024). Available from: https://openai.com/index/hello-gpt-4o/ (Accessed June 3, 2024).

[5] Bailyn E. Top Generative AI Chatbots by Market Share – December 2024. United States: First Page Sage (2024). Available from: https://firstpagesage.com/reports/top-generative-ai-chatbots/ (Accessed December 16, 2024).

[6] Newton PM Xiromeriti M. ChatGPT Performance on MCQ Exams in Higher Education. A Pragmatic Scoping Review. United States: EdArXiv (2023). Available from: https://edarxiv.org/sytu3/ (Accessed June 23, 2023).

[7] Newton PM Summers CJ Zaheer U Xiromeriti M Stokes JR Bhangu JS Can ChatGPT-4o Really Pass Medical Science Exams? A Pragmatic Analysis Using Novel Questions medRxiv (2024). Available from: https://www.medrxiv.org/content/10.1101/2024.06.29.24309595v2 (Accessed July 9, 2024).24309595 10.1007/s40670-025-02293-z PMC12058600 40352979

[8] Newton PM Da Silva A Berry S. The Case for Pragmatic Evidence-Based Higher Education: A Useful Way Forward? Front Educ (2020) 5. 10.3389/feduc.2020.583157

[9] Students O for. Degree Awarding Powers - Office for Students. Off Students (2021). Available from: https://www.officeforstudents.org.uk/ (Accessed September 9, 2024).

[10] Quality Assurance Agency. Subject Benchmark Statement - Biomedical Science and Biomedical Sciences (2023). Available from: https://www.qaa.ac.uk/the-quality-code/subject-benchmark-statements/subject-benchmark-statement-biomedical-science-and-biomedical-sciences (Accessed October 21, 2024).

[11] Quality Assurance Agency. The Frameworks for Higher Education Qualifications of UK Degree-Awarding Bodies (2024). Available from: https://www.qaa.ac.uk/docs/qaa/quality-code/the-frameworks-for-higher-education-qualifications-of-uk-degree-awarding-bodies-2024.pdf?sfvrsn=3562b281_11#:∼:text=3.4%20The%20FHEQ%20has%20five,are%20undergraduate%20and%20two%20postgraduate (Accessed October 21, 2024).

[12] Health and Care Professions Council. Standards of Education and Training (2024). Available from: https://www.hcpc-uk.org/standards/standards-relevant-to-education-and-training/set/ (Accessed October 21, 2024).

[13] Adesope OO Trevisan DA Sundararajan N. Rethinking the Use of Tests: A Meta-Analysis of Practice Testing. Rev Educ Res (2017) 87(3):659–701. 10.3102/0034654316689306

[14] Rowland CA. The Effect of Testing Versus Restudy on Retention: A Meta-Analytic Review of the Testing Effect. Psychol Bull (2014) 140(6):1432–63. 10.1037/a0037559 25150680

[15] Bloom BS Krathwohl DR. Taxonomy of Educational Objectives. In: The Classification of Educational Goals, Handbook I: Cognitive Domain (New York: Longmans Green) (1956).

[16] Newton PM Da Silva A Peters LG. A Pragmatic Master List of Action Verbs for Bloom’s Taxonomy. Front Educ (2020) 5. 10.3389/feduc.2020.00107

[17] Willingham D. How Knowledge Helps. United States: Am Fed Teach (2006). Available from: https://www.aft.org/periodical/american-educator/spring-2006/how-knowledge-helps (Accessed November 29, 2022).

[18] Race P. The Lecturers Toolkit: A Practical Guide to Assessment, Learning and Teaching. 4th ed. London: Routledge (2014). p. 300.

[19] Nikolic S Sandison C Haque R Daniel S Grundy S Belkina M ChatGPT, Copilot, Gemini, SciSpace and Wolfram Versus Higher Education Assessments: An Updated Multi-Institutional Study of the Academic Integrity Impacts of Generative Artificial Intelligence (GenAI) on Assessment, Teaching and Learning in Engineering. Australas J Eng Educ (2024) 0(0):1–28. 10.1080/22054952.2024.2372154

[20] Rossettini G Rodeghiero L Corradi F Cook C Pillastrini P Turolla A Comparative Accuracy of ChatGPT-4, Microsoft Copilot and Google Gemini in the Italian Entrance Test for Healthcare Sciences Degrees: A Cross-Sectional Study. BMC Med Educ (2024) 24(1):694. 10.1186/s12909-024-05630-9 38926809 PMC11210096

[21] Newton PM. Guidelines for Creating Online MCQ-Based Exams to Evaluate Higher Order Learning and Reduce Academic Misconduct. In: Eaton SE, Editor. Handbook of Academic Integrity. Singapore: Springer Nature (2023). p. 1–17. 10.1007/978-981-287-079-7_93-1

[22] Billings M DeRuchie K Hussie K Kulesher A Merrell J Morales A Constructing Written Test Questions for the Health Sciences. Natl Board Med Examiners (2020). Available from: https://www.nbme.org/sites/default/files/2020-11/NBME_Item%20Writing%20Guide_2020.pdf (Accessed April 7, 2022).

[23] Stribling D Xia Y Amer MK Graim KS Mulligan CJ Renne R. The Model Student: GPT-4 Performance on Graduate Biomedical Science Exams. Sci Rep (2024) 14(1):5670. 10.1038/s41598-024-55568-7 38453979 PMC10920673

[24] Newton PM Essex K. How Common Is Cheating in Online Exams and Did It Increase During the COVID-19 Pandemic? A Systematic Review. J Acad Ethics (2023) 22:323–43. 10.1007/s10805-023-09485-5

[25] Bretag T Harper R Burton M Ellis C Newton P Rozenberg P Contract Cheating: A Survey of Australian University Students. Stud High Educ (2019) 44(11):1837–56. 10.1080/03075079.2018.1462788

[26] Marano E Newton PM Birch Z Croombs M Gilbert C Draper MJ. What Is the Student Experience of Remote Proctoring? A Pragmatic Scoping Review. EdArXiv (2023). 10.1111/hequ.12506

[27] Kıyak YS Kononowicz AA. Case-Based MCQ Generator: A Custom ChatGPT Based on Published Prompts in the Literature for Automatic Item Generation. Med Teach (2024) 46, 1018, 20. 10.1080/0142159X.2024.2314723 38340312

[28] Choi W. Assessment of the Capacity of ChatGPT as a Self-Learning Tool in Medical. Pharmacol A Study Using MCQs (2023). 10.1186/s12909-023-04832-x PMC10644619 37957666

[29] Wu H Zerner T Lee D Court-Kowalski S Devitt P Palmer E. GPT-4 Versus Human Authors in Clinically Complex MCQ Creation: A Blinded Analysis of Item Quality. Res Square (2024). 10.21203/rs.3.rs-4831476/v1 40439661

[30] Artsi Y Sorin V Konen E Glicksberg BS Nadkarni G Klang E. Large Language Models for Generating Medical Examinations: Systematic Review. BMC Med Educ (2024) 24(1):354. 10.1186/s12909-024-05239-y 38553693 PMC10981304

[31] Khowaja SA Khuwaja P Dev K Wang W Nkenyereye L. ChatGPT Needs SPADE (Sustainability, PrivAcy, Digital Divide, and Ethics) Evaluation: A Review. Cogn Comput (2024) 16(5):2528–50. 10.1007/s12559-024-10285-1

[32] Scarfe P Watcham K Clarke A Roesch E. A Real-World Test of Artificial Intelligence Infiltration of a University Examinations System: A “Turing Test” Case Study. Plos One (2024) 19(6):e0305354. 10.1371/journal.pone.0305354 38923941 PMC11206930

[33] Yeadon W Inyang OO Mizouri A Peach A Testrow CP. The Death of the Short-Form Physics Essay in the Coming AI Revolution. Phys Educ (2023) 58(3):035027. 10.1088/1361-6552/acc5cf

[34] Revell T Yeadon W Cahilly-Bretzin G Clarke I Manning G Jones J ChatGPT Versus Human Essayists: An Exploration of the Impact of Artificial Intelligence for Authorship and Academic Integrity in the Humanities. Int J Educ Integr (2024) 20(1):18–9. 10.1007/s40979-024-00161-8

[35] Williams A. Comparison of Generative AI Performance on Undergraduate and Postgraduate Written Assessments in the Biomedical Sciences. Int J Educ Technol High Educ (2024) 21(1):52. 10.1186/s41239-024-00485-y

[36] Giray L. ChatGPT References Unveiled: Distinguishing the Reliable From the Fake. Internet Ref Serv Q (2024) 28(1):9–18. 10.1080/10875301.2023.2265369

[37] Sarode G Singh S Sarode SC. Cautionary Note: Chatbots Generate Fictitious References on the Topics Related to Oral Pathology. Oral Oncol Rep (2024) 9:100168. 10.1016/j.oor.2024.100168

[38] Waltzer T Cox RL Heyman GD. Testing the Ability of Teachers and Students to Differentiate Between Essays Generated by ChatGPT and High School Students. Hum Behav Emerg Technol (2023) 2023:e1923981–9. 10.1155/2023/1923981

[39] Perkins M Roe J Postma D McGaughran J Hickerson D. Detection of GPT-4 Generated Text in Higher Education: Combining Academic Judgement and Software to Identify Generative AI Tool Misuse. J Acad Ethics (2023) 22:89–113. 10.1007/s10805-023-09492-6

[40] Weber-Wulff D Anohina-Naumeca A Bjelobaba S Foltýnek T Guerrero-Dib J Popoola O Testing of Detection Tools for AI-Generated Text. arXiv (2023) 19:26. 10.1007/s40979-023-00146-z

[41] Gorichanaz T. Accused: How Students Respond to Allegations of Using ChatGPT on Assessments. Learn Res Pract (2023) 9(2):183–96. 10.1080/23735082.2023.2254787

[42] Perkins M Roe J Vu BH Postma D Hickerson D McGaughran J Simple Techniques to Bypass GenAI Text Detectors: Implications for Inclusive Education. Int J Educ Technol High Educ (2024) 21(1):53. 10.1186/s41239-024-00487-w

[43] Wallace MJ Newton PM. Turnaround Time and Market Capacity in Contract Cheating. Educ Stud (2014) 40(2):233–6. 10.1080/03055698.2014.889597

[44] Ward M O’Riordan F Logan-Fleming D Cooke D Concannon-Gibney T Efthymiou M Interactive Oral Assessment Case Studies: An Innovative, Academically Rigorous, Authentic Assessment Approach. Innov Educ Teach Int (2024) 61(5):930–47. 10.1080/14703297.2023.2251967

[45] Misra SM Suresh S. Artificial Intelligence and Objective Structured Clinical Examinations: Using ChatGPT to Revolutionize Clinical Skills Assessment in Medical Education. J Med Educ Curric Dev (2024) 11:23821205241263475. 10.1177/23821205241263475 39070287 PMC11273588

[46] Holderried F Stegemann–Philipps C Herschbach L Moldt JA Nevins A Griewatz J A Generative Pretrained Transformer (GPT)–Powered Chatbot as a Simulated Patient to Practice History Taking: Prospective, Mixed Methods Study. JMIR Med Educ (2024) 10(1):e53961. 10.2196/53961 38227363 PMC10828948

[47] Pereira DSM Falcão F Nunes A Santos N Costa P Pêgo JM. Designing and Building OSCEBot ® for Virtual OSCE - Performance Evaluation. Med Educ Online (2023) 28(1):2228550. 10.1080/10872981.2023.2228550 37347808 PMC10288924

[48] Brannick MT Erol-Korkmaz HT Prewett M. A Systematic Review of the Reliability of Objective Structured Clinical Examination Scores. Med Educ (2011) 45(12):1181–9. 10.1111/j.1365-2923.2011.04075.x 21988659

[49] Health and Care Professions Council. Standards of Proficiency for Biomedical Scientists (2024). Available from: https://www.hcpc-uk.org/standards/standards-of-proficiency/biomedical-scientists/ (Accessed October 21, 2024).

[50] HCPC. Release of Revised Standards of Conduct, Performance and Ethics (2023). Available from: https://www.hcpc-uk.org/news-and-events/news/2023/release-of-revised-standards-of-conduct-performance-and-ethics/ (Accessed December 17, 2024).

[51] Institute for Biomedical Science. Registration Training Portfolio (2024). Available from: https://www.ibms.org/education/registration-portfolio/ (Accessed October 21, 2024).

[52] Plesner LL Müller FC Brejnebøl MW Krag CH Laustrup LC Rasmussen F Using AI to Identify Unremarkable Chest Radiographs for Automatic Reporting. Radiology (2024) 312(2):e240272. 10.1148/radiol.240272 39162628

[53] Klauschen F Dippel J Keyl P Jurmeister P Bockmayr M Mock A Toward Explainable Artificial Intelligence for Precision Pathology. Annu Rev Pathol Mech Dis (2024) 19:541–70. 10.1146/annurev-pathmechdis-051222-113147 37871132

